# Systemic Infection of* Nicotiana benthamiana* with* Potato virus X* Nanoparticles Presenting a Fluorescent iLOV Polypeptide Fused Directly to the Coat Protein

**DOI:** 10.1155/2018/9328671

**Published:** 2018-02-13

**Authors:** Juliane Röder, Christina Dickmeis, Rainer Fischer, Ulrich Commandeur

**Affiliations:** Institute for Molecular Biotechnology, RWTH Aachen University, Worringerweg 1, 52072 Aachen, Germany

## Abstract

Plant virus-based nanoparticles can be produced in plants on a large scale and are easily modified to introduce new functions, making them suitable for applications such as vaccination and drug delivery, tissue engineering, and* in vivo* imaging. The latter is often achieved using green fluorescent protein and its derivatives, but the monovalent fluorescent protein iLOV is smaller and more robust. Here, we fused the iLOV polypeptide to the N-terminus of the* Potato virus X* (PVX) coat protein, directly or via the* Foot-and-mouth disease virus* 2A sequence, for expression in* Nicotiana benthamiana*. Direct fusion of the iLOV polypeptide did not prevent the assembly or systemic spread of the virus and we verified the presence of fusion proteins and iLOV hybrid virus particles in leaf extracts. Compared to wild-type PVX virions, the PVX particles displaying the iLOV peptide showed an atypical, intertwined morphology. Our results confirm that a direct fusion of the iLOV fluorescent protein to filamentous PVX nanoparticles offers a promising tool for imaging applications.

## 1. Introduction

The functionalization of plant virus-based nanoparticles is an emerging field, facilitated by the well-characterized genomic sequence and modular organization of many different plant viruses [1–3]. Plant viruses come in various shapes and sizes with different surface properties, allowing researchers to pick the most suitable virus for a given application. The structures of many plant viruses are known to atomic or near-atomic resolution [4–7]. The advantages of plant virus-based nanoparticles include their ability to self-assemble with precise symmetry and polyvalency, their monodispersity and stability under a range of conditions, their inherent safety (biocompatibility and noninfectivity in mammals), and the rapid, simple and scalable production that can be achieved by molecular farming in plants [8]. None of these properties can be matched by synthetic nanoparticles.

*Potato virus X* (PVX) is the type member of the genus* Potexvirus* (family* Alphaflexiviridae*) and is often used as a vector for heterologous gene expression in plants [9–18]. The complete 6.4 kb genomic RNA sequences of several PVX strains are known [19–21]. The plus-strand RNA genome has a 5′-methylguanosine cap and is polyadenylated at the 3′-terminus [22, 23]. Each PVX particle comprises ~1270 coat protein (CP) subunits helically arranged to form filamentous, flexible virions encapsidating the viral genomic RNA. The C-terminus of each CP subunit is located within the PVX particle whereas the N-terminus is exposed on the surface, providing an ideal site for the presentation of peptides and proteins [4, 24–28].

Genetic modification or chemical conjugation can be used to tailor the surface properties of plant viruses allowing them to be engineered, for example, as drug delivery vehicles, tissue scaffolds, and electronic devices [1, 8, 29–32]. Furthermore, fluorescence-labeled virus nanoparticles can be used for biomedical imaging [33–39]. Such labeling is usually achieved by the conjugation of chemical dyes to functional groups on the virus surface after particle assembly. Several studies have shown that PVX particles carrying molecular contrast agents such as Alexa Fluor647 can efficiently penetrate solid tumors and accumulate inside [40–42]. However, chemical conjugation results in a limited number of tagged CP subunits, whereas the genetic fusion of a fluorescent protein to the CP allows denser labeling [35]. The latter strategy can be challenging because the size and biochemical properties of the fusion protein may interfere with particle assembly, stability, or infectivity. Single amino acids or peptides can be fused directly to the N-terminus of the PVX CP, whereas longer peptides that prevent particle assembly require the presence of wild-type PVX CPs to avoid steric hindrance, resulting in the formation of hybrid particles containing both wild-type CP and the fusion protein. This is generally achieved by inserting the* Foot-and-mouth disease virus* (FMDV) 2A sequence between the transgene and the* cp* sequence, yielding a CP fusion protein, free target protein, and wild-type CP due to a ribosomal skip during translation (the overcoat method) [43–45]. The biochemical properties of peptides and proteins fused to the CP can also affect virus particle assembly, stability, or functionality (e.g., systemic spreading), including the number of serine/threonine [46] or tryptophan residues [47], and the isoelectric point (pI) [47, 48]. A PVX-mCherry overcoat system for* in vivo* tumor imaging in mouse models was recently reported [37].

Although fluorescent proteins such as mCherry and green fluorescent protein (GFP) are routinely used as reporters for molecular interactions, they are dependent on pH and oxygen, and their large size (at least ~240 amino acids) and complex structure make them more difficult to express [49–51]. The recently discovered monomeric fluorescent protein iLOV (light, oxygen, or voltage sensing) was engineered from the LOV2 domain of* Arabidopsis thaliana* phototropin  2 and can be fused to the movement protein of TMV for quantitative biological imaging applications [52–55]. The iLOV sequence is approximately half the size of mCherry thus reducing the genetic load on the recombinant viruses, and its reversible photobleaching properties make it ideal for the analysis of virus movement in plants [52, 55, 56]. Here, we compared the behavior of PVX-iLOV particles created by either direct fusion or the use of the FMDV 2A sequence, focusing on their long-distance movement and propensity for systemic infection and the morphology of the modified particles compared to wild-type PVX.

## 2. Materials and Methods

### 2.1. Construction of Plant Viral Vectors

**Figure 1:**
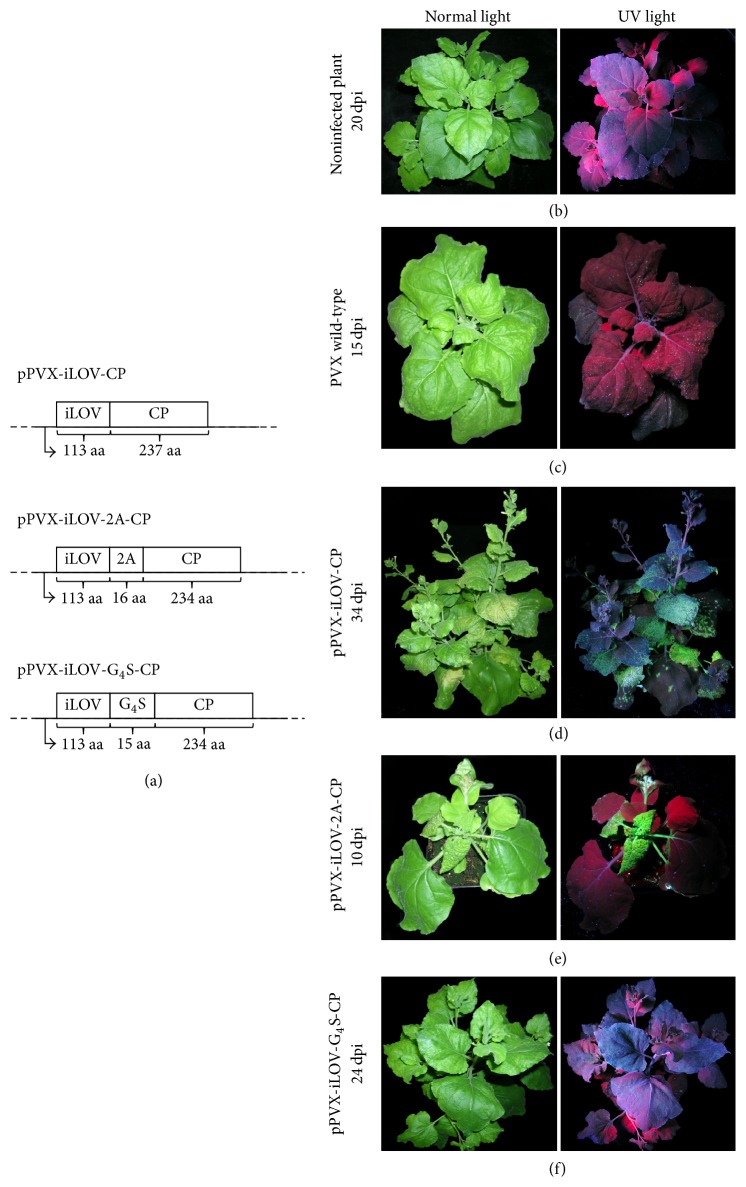
PVX-derived vectors for the expression of iLOV and the infection of* N. benthamiana*. (a) The iLOV coding sequence was added either as a direct fusion to the 5′-end of the PVX coat protein (CP) gene, with a (G_4_S)_3_ linker, or as a fusion to the FMDV 2A sequence. The target genes are depicted as part of the complete viral genome. (b–f) Symptoms and detection of fluorescence in* N. benthamiana* plants inoculated with hybrid PVX. (b) Noninfected* N. benthamiana* plant and plants infected with (c) wild-type PVX, (d) pPVX-iLOV-CP, (e) pPVX-iLOV-2A-CP, and (f) pPVX-iLOV-G_4_S-CP were displayed either under UV light or under normal light 10–34 days after inoculation (dpi) depending on the progress of infection.

The PVX-based vectors we used to produce recombinant particles displaying the iLOV polypeptide are shown in Figure 1(a). The iLOV sequence was amplified from source vector pSC1001a (Dr. S. Chapman, The James Hutton Institute, Dundee, Scotland) using the primers listed in Table 1 and was transferred to pCR2.1-TOPO as previously described [57]. An iLOV fragment was isolated from this intermediate vector using NheI and BspEI and was transferred to vector pTCXIIc [37] either as a direct fusion (replacing the mCherry and FMDV 2A genes) or as fusion via the FMDV 2A sequence (replacing only the mCherry gene). The iLOV sequence was also inserted into pPVX-N-mCherry-G_4_S-CP (our unpublished data) to create an iLOV-CP fusion via a (Gly_4_Ser)_3_ linker (G_4_S). The integrity of the resulting vectors pPVX-iLOV-CP, pPVX-iLOV-2A-CP, and pPVX-iLOV-G_4_S-CP was tested by PCR and sequencing. The vectors were propagated in* Escherichia coli *strain DH5*α* ready for the inoculation of* Nicotiana benthamiana* plants as previously described [57].

### 2.2. RNA and Protein Analyses

Total RNA was isolated from systemically infected* N. benthamiana* leaves using the RNeasy Plant Mini Kit (Qiagen, Hilden, Germany) and 3 *μ*g RNA samples were treated with 3 U DNaseI (Thermo Fisher Scientific) as recommended. The corresponding cDNA was produced using M-MLV Reverse Transcriptase RNaseH Minus Point Mutant (Promega, Madison, USA). We mixed 1 *μ*g of the DNaseI-treated RNA with 0.5 *μ*M of the oligo-dT primer for hybrid PVX particles and 27 *μ*l DEPC-treated water. The mixture was heated to 80°C and then incubated for 10 min on ice to ensure primer annealing before adding 10 *μ*l 5x M-MLV reaction buffer, 8.5 *μ*l DEPC-treated water, 1 mM dNTPs, and 1 *μ*l M-MLV. The cDNA synthesis reaction was carried out for 30 min at 40°C, 20 min at 45°C, 20 min at 50°C, 20 min at 55°C, and 20 min at 70°C. After reverse transcription, residual RNA was degraded with 1 *μ*l 10 mg/ml RNaseA for 1 h at 37°C. PCR was carried out with primers TGB-fw and CX4 and the products were resolved by 1.2% agarose gel electrophoresis to confirm the integrity of the iLOV-CP and iLOV-2A-CP cDNAs.

RNA levels in PVX-infected leaves were determined by quantitative real-time PCR (qPCR). We mixed 5 *μ*l of recombinant PVX cDNA samples with 0.3 *μ*M each of primers CX4i and CX8, 10 *μ*l 2x iQ™ SYBR Green® supermix (BioRad, Munich, Germany), and 3.8 *μ*l water. The qPCR program comprised three steps: 3 min at 95°C for polymerase activation and DNA denaturation and then 40 cycles of 15 s at 95°C, 30 s at 55°C, and 45 s at 72°C measured with SYBR Green. Finally, melting curves were constructed from 55°C to 95°C in 0.5°C/2 s steps using primers for the* cp* (CX4i and CX8) and* tgb* (CX3i and CX9) as previously described [14]. Triplicate measurements were taken with samples from at least three different plants.

Proteins were extracted from systemically infected* N. benthamiana* leaves and were analyzed by SDS-PAGE [58] and western blot, with specific antibodies against iLOV and the PVX CP as previously described [57].

### 2.3. Confocal Microscopy

Systemic infection with recombinant PVX particles was visualized by confocal laser scanning microscopy using a Leica TCS SP instrument (Leica Microsystems, Wetzlar, Germany). We removed 1 × 1 cm leaf pieces showing fluorescence and mounted them with the abaxial surface facing upwards onto glass microscope slides. An HCX PL APO 63.0 × 5.00 water-corrected objective was used with a drop of water placed directly on the coverslip to image viral cell-to-cell movement across the epidermal cell layer. The excitation and detection wavelengths for iLOV were 476 nm and 510–550 nm, respectively. LCS software was used for image analysis, following import into Photoshop CS5 (Adobe Systems, Mountain View, USA) for contrast adjustments and overlay.

### 2.4. Purification of iLOV-Displaying Particles

Purified iLOV-CP and iLOV-2A-CP virus particles were prepared as described in the protocol published by the International Potato Centre (Lima, Peru) with modifications as previously described [13]. Centrifugation with a sucrose cushion was omitted and the pellet was directly resuspended after clarification. Subsequent centrifugation was carried out at 7800 ×g for 10 min at 4°C instead of 2000 ×g for 5 min at 4°C, and the second ultracentrifugation to sediment the virions was at least 3-4 h instead of 1 h. The final clarification step was also omitted.

### 2.5. Transmission Electron Microscopy

Immunosorbent electron microscopy (ISEM) was carried out with virus-containing plant extracts from systemically infected leaves or purified recombinant viruses. Pioloform-coated nickel grids (Plano, Wetzlar, Germany) were incubated on a drop of monoclonal antibody MAC58 against PVX (kindly provided by Professor L. Torrance, The James Hutton Institute) for 20 min at room temperature to capture the recombinant PVX particles. Immunogold decoration of captured or adsorbed particles with primary anti-PVX antibody or the anti-iLOV antiserum and secondary goat anti-rabbit (GAR) 15 nm gold conjugate (British BioCell, Cardiff, UK) and imaging was carried out as previously described [57].

## 3. Results

### 3.1. Vector Construction and Infection of Plants

Three different PVX-based expression constructs were prepared, joining the iLOV sequence to the 5′-end of the PVX* cp* gene using different strategies: (a) a direct fusion, (b) via a (Gly_4_Ser)_3_ linker, and (c) via the FMDV 2A sequence (Figure 1(a)). Following the inoculation of* N. benthamiana* plants separately with each vector, plants infected with pPVX-iLOV-2A-CP showed initial fluorescent spots at the inoculation site 4 days after inoculation (dpi) and systemic infection was observed 6 dpi, as revealed by the general green fluorescence. These plants also displayed more severe symptoms than typically observed for systemic infection with wild-type PVX, including mosaic patterns and dwarfing, and necrotic lesions appearing from 10 dpi onwards (Figures 1(c) and 1(e)). In contrast, plants infected with pPVX-iLOV-CP showed initial fluorescent spots at the inoculation site 8 dpi or later. The long-distance movement of the virus was significantly delayed: systemic infection was not observed until 17–21 dpi (Figure 1(d)). Plants inoculated with the pPVX-iLOV-G_4_S-CP vector did not show any signs of systemic infection (Figure 1(f)).

**Figure 2:**
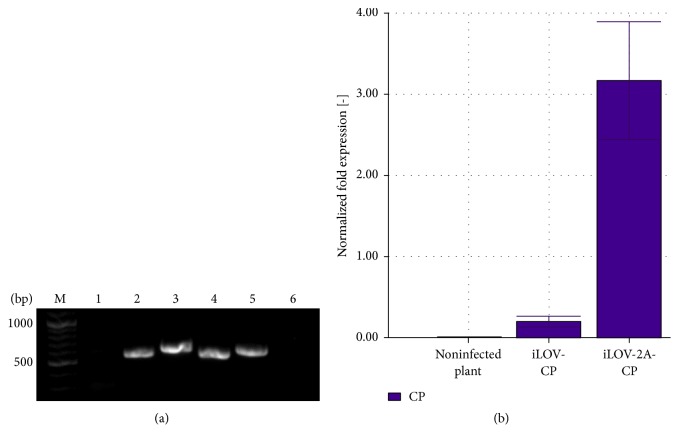
Analyses of RNA levels in systemically infected* N. benthamiana* leaves by reverse transcription PCR and qPCR. (a) Reverse transcription PCR of isolated RNA used for qPCR. M: Gene Ruler 100 bp Plus DNA ladder (Thermo Fisher Scientific), 1: negative control, RNA from noninfected* N. benthamiana*, 2: pPVX-iLOV-CP infected, 3: pPVX-iLOV-2A-CP infected, 4: positive control, plasmid pPVX-iLOV-CP, 5: positive control, plasmid pPVX-iLOV-2A-CP, and 6: negative control, DEPC-treated water. (b) qPCR analysis. Normalized fold expression is shown in relation to the* tgbp3* and* cp* genes. Measurements were performed in triplicate and repeated with samples from at least three different plants. Error bars show standard deviations of the 2^−ΔΔ*C*_*T*_^ values.

Quantitative real-time PCR (qPCR) was carried out to determine the levels of* cp* RNA in leaves from systemically infected* N. benthamiana* plants (Figure 2). Total RNA was isolated from leaves showing iLOV fluorescence, cDNA synthesis was carried out and the integrity of the modified PVX sequence was verified by a control PCR with primers TGB-fw and CX4, amplifying a region between the triple gene block protein 3* (tgbp3)* and* cp* genes (Figure 2(a)). We then determined the relative levels of the* tgbp3* and* cp* RNAs with the housekeeping gene* PP2A* used as a reference. The amount of* ilov *and* cp *RNA was compared to the amount of* tgb3* RNA, which can be found in the PVX genomic RNA (gRNA) and subgenomic RNAs (sgRNAs) 1 and 2 but not sgRNAs 3. This allowed a direct correlation between the iLOV-(2A)-CP RNA level and the total amount of PVX RNA. The quantity of* tgbp3* and* cp* RNA was correlated to* PP2A* mRNA and the relative amount of* cp* to* tgb3* was calculated and compared. We observed higher relative* cp* RNA levels for the iLOV-2A-CP fusion than for the iLOV-CP direct fusion (Figure 2(b)). This was consistent with the rapid infection and severe symptoms in plants infected with pPVX-iLOV-2A-CP compared to the slower systemic infection with pPVX-iLOV-CP.

### 3.2. Analysis of Recombinant PVX Particles Displaying iLOV

**Figure 3:**
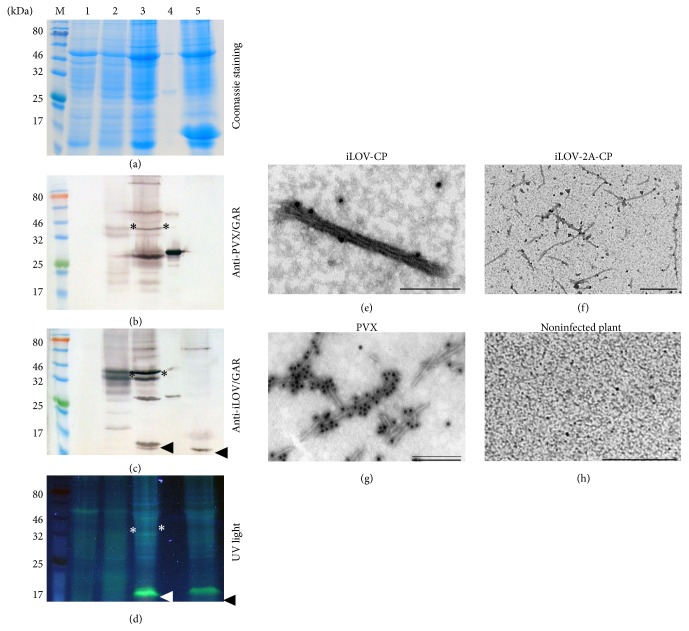
Analyses of PVX-infected* N. benthamiana* leaves expressing iLOV. (a–d) Leaf extracts (20 *μ*l) were separated by SDS-PAGE and proteins were (a) stained with Coomassie Brilliant Blue, (b, c) transferred onto a nitrocellulose membrane for western blotting, or (d) detected under UV light. M: P7712 Prestained Protein Standard (NEB), 1: negative control, plant extract from noninfected* N. benthamiana* leaves, 2: plant extract from pPVX-iLOV-CP infected leaves, 3: plant extract from pPVX-iLOV-2A-CP infected leaves, 4: positive control, PVX particles, and 5: positive control, plant extract of iLOV expressed by TMV-derived vector under control of a subgenomic promoter-like sequence [57]. Antibody detection: (b) anti-PVX and GAR^AP^ and (c) anti-iLOV and GAR^AP^. iLOV-CP and iLOV-2A-CP fusion proteins are highlighted with asterisks, and the free iLOV polypeptide is highlighted with an arrow. (e–h) Electron micrographs of leaf extracts expressing recombinant PVX particles displaying iLOV. (e) iLOV-CP, (f) iLOV-2A-CP, and (g) PVX particles captured with MAC58, detected with anti-PVX, and labeled with GAR 15 nm gold conjugate. Bar = (e) 200 nm, (f, g) 500 nm, and (h) 1 *μ*m.

Extracts were prepared from leaves containing the pPVX-iLOV-CP and pPVX-iLOV-2A-CP fusion proteins for analysis by SDS-PAGE and western blot. The iLOV-CP and iLOV-2A-CP fusion proteins were detected with the anti-PVX antibody and the anti-iLOV antiserum with molecular weights of 38.0 and 39.9 kDa, respectively, as expected (Figures 3(b) and 3(c) highlighted with asterisks). A 25 kDa band representing the wild-type PVX CP was also detected in the samples containing iLOV-2A-CP because fusion to the FMDV 2A sequence is known to cause a ribosomal skip (Figure 3(b)). However, the CP was also detected by the anti-iLOV antiserum (Figure 3(c)). The 13.5 kDa free iLOV protein (highlighted with an arrow) was also visible in the western blot probed with anti-iLOV antiserum (Figure 3(c)) as well as in the polyacrylamide gel under UV light (Figure 3(d)). Densitometric analysis of iLOV bands in the anti-iLOV western blot showed a ratio of approximately 7 to 3 for free iLOV to the iLOV-2A-CP fusion protein.

The ability of the fusion proteins to assemble into PVX particles was confirmed by electron microscopy. PVX particles were immunocaptured from leaf extracts using monoclonal antibody MAC58, detected with a primary anti-PVX antibody and decorated with GAR 15 nm gold conjugates (Figures 3(e)–3(h)). Typically, wild-type PVX particles are flexible 515 × 13.5 nm structures (Figure 3(g)), but iLOV-CP and iLOV-2A-CP particles showed a different morphology (Figures 3(e) and 3(f)). These particles were of the same size as wild-type particles but were intertwined, resulting in a morphology similar to that described for PVX particles with N-terminal coat protein truncations [59].

### 3.3. Localization of iLOV within the Plant Cell

**Figure 4:**
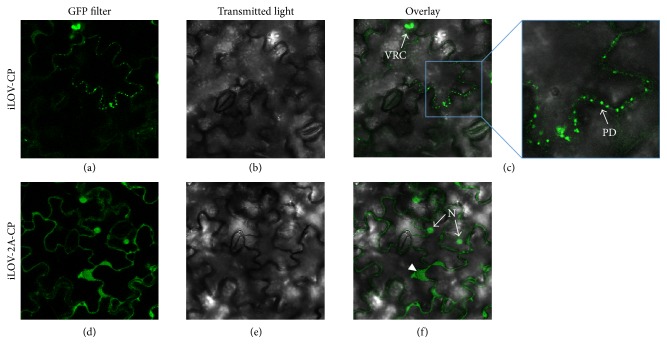
Detection of green fluorescence in epidermal cells from* N. benthamiana* leaves systemically infected with pPVX-iLOV-CP and pPVX-iLOV-2A-CP. The iLOV fluorescence was observed by confocal laser scanning microscopy. The hybrid particles indicate cell-to-cell movement at plasmodesmata (PD) and viral replication complexes (VRCs). For iLOV-2A-CP there is also free iLOV visible in the cytoplasm (▸). N: nucleus.

To gain insight into the cell-to-cell movement of hybrid PVX particles, leaves systemically infected with pPVX-iLOV-CP and pPVX-iLOV-2A-CP were studied by confocal laser scanning microscopy (Figure 4). The iLOV fluorescence was concentrated in certain areas of the cell wall, which potentially indicates the cell-to-cell movement of PVX-iLOV particles through the plasmodesmata. The fluorescence profile (Figures 4(c) and 4(f)) indicated that the virus uses these intercellular links to cross the plant cell wall [60, 61]. We also observed large viral replication complexes (VRCs) within cells, typically one per infected cell [62, 63]. In leaves infected with pPVX-iLOV-2A-CP, we detected free iLOV protein in the cytoplasm and in the nucleus, reflecting the ribosomal skip at the FMDV 2A sequence (Figure 4(f)). The fluorescence of the iLOV-CP direct fusion led to a more precise localization of the hybrid virus within the plasmodesmata.

### 3.4. Purification and Immunogold Labeling of Virus Particles

**Figure 5:**
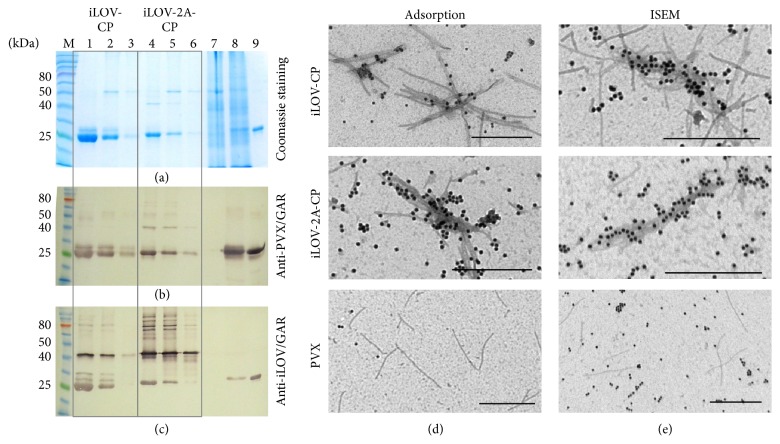
Analyses of purified iLOV-CP and iLOV-2A-CP particles. (a–c) Purified PVX particles (5 *μ*g) displaying iLOV separated by SDS-PAGE. M: P7711 Prestained Protein Standard (NEB), 1: iLOV-CP sample 1, 2: iLOV-CP sample 2, 3: iLOV-CP sample 3, 4: iLOV-2A-CP sample 1, 5: iLOV-2A-CP sample 2, 6: iLOV-2A-CP sample 3, 7: negative control, noninfected* N. benthamiana* leaf extract, 8: PVX-GFP-2A-CP infected leaf extract, and 9: positive control, PVX particles. Antibody detection: (b) anti-PVX and GAR^AP^ and (c) anti-iLOV and GAR^AP^. (d–e) Transmission electron microscopy of recombinant particles. Adsorption: detection with anti-iLOV and decoration with GAR 15 nm gold conjugate, ISEM: capture with monoclonal MAC58, detection with anti-iLOV, and decoration with GAR 15 nm gold conjugate. Bar = 500 nm.

PVX particles displaying iLOV were purified with yields of 136–206 mg per kg of infected leaf material by adapting the purification protocol for wild-type PVX particles developed by the International Potato Centre. The different pooled fractions were tested for the presence of iLOV-CP and iLOV-2A-CP by western blot (Figure 5). Both the polyclonal anti-PVX antibody and the anti-iLOV antiserum detected some degradation products but also intact proteins with the anticipated molecular weights of the iLOV-CP and iLOV-2A-CP fusion proteins (Figures 5(b) and 5(c)). The iLOV-CP fusion protein is only detected in very low amounts with an anti-PVX antibody, probably due to the low amounts of purified particles and protein degradation. The anti-iLOV antiserum also detected bands of the wild-type PVX CP, and thus it was necessary to find out whether the antiserum was able to also detect wild-type PVX particles by electron microscopy. Purified iLOV-CP, iLOV-2A-CP, and wild-type PVX virions were directly adsorbed onto the grids (Figure 5(d)) or captured using monoclonal antibody MAC58 (Figure 5(e)). The fusion proteins were detected by the primary anti-iLOV antiserum and labeled with the secondary GAR 15 nm gold conjugate. We found that only PVX particles displaying the iLOV protein on the surface were labeled, not the wild-type PVX particles. Thus, scattered single gold particles are most likely background and not bound to particles. These experiments also confirmed that the heterologous fusion protein is accessible even if the recombinant virions are intertwined.

## 4. Discussion

The biological compatibility and multivalency of genetically and chemically addressable sites on virus CPs have resulted in a broad range of applications for plant virus nanoparticles. In this study, PVX-derived vectors (Figure 1(a)) were generated to present the monomeric fluorescent protein iLOV on the surface of PVX particles by fusing it to the 5′-terminus of the PVX CP directly, via a FMDV 2A sequence (overcoat), or via a sequence coding for a flexible glycine-serine-rich linker. The direct fusion (pPVX-iLOV-CP) and overcoat (pPVX-iLOV-2A-CP) constructs were able to achieve systemic infection whereas the linker construct (pPVX-iLOV-G_4_S-CP) was not, most likely due to size limitation [15, 48, 64, 65], and the latter was therefore excluded from further experiments. To exclude the possibility that the flexibility of the G_4_S-linker enables iLOV to move between the CP subunits and prevents particle assembly, we replaced the G_4_S linker with the more rigid AK_3_A linker (three copies of the sequence AKKKA). The plants showed neither green fluorescence in inoculated leaves nor systemic infection at 28 dpi (data not shown).

Systemic infection with pPVX-iLOV-CP was delayed and the infection symptoms were mild, whereas rapid systemic infection was established following inoculation with pPVX-iLOV-2A-CP and the infection symptoms were severe, including necrosis (Figures 1(d) and 1(e)). This may reflect the ability of the iLOV protein to promote the synthesis of reactive oxygen species [66]. Following inoculation with pPVX-iLOV-2A-CP, the iLOV polypeptide was overexpressed as a fusion protein but also as a free protein due to the overcoat principle, potentially triggering the accumulation of greater quantities of reactive oxygen species in these plants compared to those infected with pPVX-iLOV-CP (Figure 1(e)).

When certain peptides or proteins are fused to plant virus CPs, the assembly of the virion is sterically or electrostatically inhibited and systemic movement prevented [15, 48, 64, 65]. However, fusing the 113-amino-acid iLOV polypeptide directly to the N-terminus of the PVX CP still allows the assembly of hybrid virions which can move from cell-to-cell and achieve systemic infections via translocation through the vascular system. The iLOV polypeptide is much larger than the largest peptides that have been displayed by direct CP fusion in other plant viruses, for example, 23 amino acids for* Tobacco mosaic virus* (TMV) [64], 30 amino acids for* Plum pox virus* [67], and 50 amino acids for* Cowpea mosaic virus* [65, 68]. With the help of a 15-residue linker, a 133-amino-acid fragment of staphylococcal protein A has been displayed on the surface of TMV [69], whereas the largest peptide expressed on the surface of PVX until the current report was 14 amino acids in length [12]. Fusion peptides larger than 40 amino acids usually require the presence of the wild-type PVX CP to assemble into intact hybrid particles, which is most easily achieved by incorporating the FMDV 2A sequence [43] as shown by the successful display of the 45 kDa VP6 rotavirus capsid protein (397 amino acids) on PVX [11]. The ratio of fusion protein to wild-type CP cannot be predicted accurately [70, 71], although using different variants of 2A sequences provides a degree of control [71]. As a tool for the bioimaging of viral cell-to-cell movement, fluorescent proteins should be densely arrayed on the virus surface to achieve a bright signal, as shown for the iLOV-CP direct fusion but not for the iLOV-2A-CP construct, which also generated diffuse fluorescence mainly caused by the free iLOV polypeptide (Figure 4).

We investigated the* cp* RNA level in relation to the* tgbp3* RNA by qPCR because the PVX* tgbp3* sequence is found on the gRNA and all sgRNAs except that carrying the* cp* gene, whereas the* cp* sequence is found on the gRNA and all sgRNAs. The comparison of* cp* and* tgbp3* RNA levels therefore provides an estimation of* cp* RNA levels with a built-in internal control. The level of iLOV-CP RNA was lower than that of iLOV-2A-CP RNA (Figure 2), explaining why the iLOV-CP particles spread more slowly through the plant. Our data suggest there are lower levels of sgRNA3 and thus lower levels of the iLOV-CP fusion protein, which can assemble to intact particles for long-distance movement. A certain ratio between the number of assembled particles and the quantity of free PVX CP [46] is necessary for viral cell-to-cell movement and long-distance transport [47, 61, 72–74]. This is why fewer iLOV-CP particles assembled compared to iLOV-2A-CP particles, thus reducing the viral load. These results are consistent with the observed delayed systemic spread of iLOV-CP particles and the rapid infection and severe symptoms caused by iLOV-2A-CP. However, the differences of sgRNA levels are unexpected, given that the iLOV sequence is incorporated into the viral genome in the same way in both constructs. The only difference is the 2A sequence introduced between the iLOV and CP sequences. The introduction of the 2A sequence in this construct therefore seems to be more favorable even for sgRNA levels.

Some PVX coat proteins with N-terminal extension peptides cannot form particles due to steric inhibition [43], the number of serine/threonine [46] or tryptophan residues [47], or the pI of the fusion peptide [47, 48]. If fusion peptides have a pI value in the range 5.2–9.2, the resulting particles can move systemically [47], although basic amino acids reduce the fitness of the virus resulting in the selection of better performing mutants [48]. Only ~13% of the amino acids in the iLOV peptide are positively charged and the iLOV-CP fusion has a pI of 5.8. There was no reversion to the wild-type PVX* cp* sequence at 34 dpi as reported for iLOV fusions to the TMV CP [57]. However, we observed proteolytic degradation of the purified iLOV-CP particles in western blots probed with the polyclonal anti-PVX antibody and anti-iLOV antiserum after particle purification (Figures 5(b) and 5(c)). The particles might be degraded during the purification process due to a lower stability of the particles in general. However, the extracts contained larger quantities of the fusion products than the virus purification (compare Figures 3(b) and 3(c) and 5(b) and 5(c)). In addition to protein degradation, we might have lost particles with a high ratio of iLOV-CP fusion to wild-type CP during the gradient purification. Particles with a greater proportion of fusion proteins accumulate in the gradient with a lower sucrose concentration and might be lost due to low visibility in the SDS gels of the gradient fractions. This could probably be prevented by adapting the purification protocol accordingly.

The presence of iLOV-decorated PVX particles in systemically infected leaves was confirmed by western blots and transmission electron microscopy (Figures 3 and 5). Wild-type PVX particles are filamentous rods, 515 nm in length—they are flexible but exist as discrete units. In contrast, the iLOV-CP and iLOV-2A-CP particles were similar in structure but the filaments tended to intertwine. A similar morphology was reported when PVX particles were assembled from N-terminally truncated CPs lacking codons 7–31 of the* cp* gene [59]. These results show that the N-terminus is required to modulate intramolecular and/or intermolecular interactions. Approximately 37% of the residues in the iLOV protein are hydrophobic, so intermolecular hydrophobic interactions may be responsible for the intertwined morphology.

Confocal laser scanning microscopy revealed that the recombinant iLOV-CP and iLOV-2A-CP particles were potentially able to pass between epidermal cells, as indicated by the presence of a fluorescent signal in the plasmodesmata (Figure 4). Similarly localized CPs with 2A fusions of fluorescent proteins have been reported in PVX movement studies [60, 61, 75]. We also observed one large fluorescent VRC per infected cell, which is frequently observed in established infections [60, 62, 63]. These so-called “virus factories” coordinate the infection processes [62]. Additional diffuse fluorescence was observed in epidermal cells infected with the pPVX-iLOV-2A-CP construct, representing the presence of free iLOV polypeptide. Free iLOV was also detected in western blots, with a ratio of 7 to 3 relative to the fusion protein. This leads to a high background of free iLOV in the cells, preventing the detailed analysis of CP localization. These findings emphasize the advantage of direct fusions of fluorescent proteins to the target protein.

Fluorescent proteins can provide insight into many cellular processes and interactions but also suffer from drawbacks such as their complex structure, large size, pH, and oxygen dependence [50, 51]. The iLOV polypeptide has spectral characteristics similar to GFP [76], but it is monomeric, has a lower molecular weight, is more photostable, and undergoes reversible photobleaching, the latter providing advantages for imaging applications [52, 55]. The presentation of the iLOV fluorescent protein on the surface of filamentous PVX offers a promising new imaging tool, particularly for the investigation of PVX infections. More specifically, these hybrid virions may be able to provide insight into the subcellular location of CP synthesis and the distribution and packaging of gRNA. The iLOV polypeptide can also be used for antiviral drug screening [77].

## 5. Conclusion

We have demonstrated that the 113-amino-acid fluorescent protein iLOV can be directly fused to the N-terminus of the PVX coat protein without the help of a flexible linker and is displayed on the particle surface. Because the iLOV protein has several advantages over other fluorescent proteins, it will be useful not only for the analysis of VRCs and long-distance virus movement, which currently relies on the production of overcoat structures, but also as a new platform for the molecular imaging of tumors. We recently reported an mCherry PVX overcoat system for tumor imaging in mouse models [37]. Our PVX-iLOV particles generated a strong fluorescent signal due to the dense array of iLOV polypeptides, offering a highly sensitive new modality for imaging applications.

## Figures and Tables

**Table 1:** Oligonucleotides used for PCR and cDNA synthesis.

Primer name	Nucleotide sequence (5′→3′)
NheI-iLOV	AAAGCTAGCATGGCAAGCATAGAGAAGAA
iLOV-BspEI	TTTTCCGGATACATGATCACTTCCATCGA
iLOV-Stop-Not	TTTGCGGCCGCTTATACATGATCACTTCCATCGAGC
M13 rev	ACACAGGAAACAGCTATGAC
M13 fw	GTTGTAAAACGACGGCCAGT
TGB-fw	AAGGGCCATTGCCGATCTCAAGC
CX1	TTGAAGAAGTCGAATGCAGC
CX3i	GAAGTGCTAATGACTGCTAT
CX4	CGGGCTGTACTAAAGAAATC
CX4i	GATTTCTTTAGTACAGCCCG
CX8	AGCTCTGCTGATGCCGTTGG
CX9	ACACGGAGGAGCTTACAGAG
